# Severe Immune Thrombocytopenic Purpura Following Influenza Vaccination: A Case Report

**DOI:** 10.7759/cureus.21250

**Published:** 2022-01-14

**Authors:** Ryuichi Ohta, Chiaki Sano

**Affiliations:** 1 Communiy Care, Unnan City Hospital, Unnan, Shimane, JPN; 2 Community Medicine Management, Shimane University Faculty of Medicine, Izumo, JPN

**Keywords:** petechiae, rural hospitals, hemorrhage, older individuals, influenza vaccine, immune thrombocytopenia

## Abstract

Immune thrombocytopenia (ITP) is a rare, acquired bleeding disorder caused by various underlying etiologies. ITP can be triggered by medication, infections, cancers, and autoimmune diseases. One of the rare triggers is vaccination. As in many cases, the symptoms are mild and the cause is idiopathic, and it may not be diagnosed without extensive investigations. It can also be difficult to differentiate between medication-induced thrombocytopenia and ITP, particularly in older patients with multiple comorbidities and receiving multiple medications. Here, we describe an older patient with acute onset ITP following influenza vaccination.

An 88-year-old man presented with complaints of systemic itchiness and bleeding from his mouth. Four days prior to the symptoms appearing, he had received an influenza vaccine and did not experience any severe pain or anaphylactic symptoms. On presentation, he had multiple bleeding blisters and systemic petechiae. Blood tests revealed a platelet count of 1000/µL. A month prior, his platelet count was 1.5×10^5^/µL. The blood culture results were negative and a bone marrow biopsy revealed multiple megakaryocytes, without blastocytes or evidence of hemophagocytosis. The patient was diagnosed with influenza vaccine-induced severe ITP and was treated with intravenous high-dose prednisolone. Although vaccine-related ITP is rare, in patients with systemic symptoms, primary care physicians should perform systematic physical examinations to detect any hemorrhage, as ITP is a possibility.

## Introduction

Immune thrombocytopenia (ITP) is a rare, acquired bleeding disorder that may be attributable to various underlying disorders. Generally, it is not severe and has mild symptoms [1]. Approximately 20-33% of patients with ITP are asymptomatic at the time of diagnosis [1]. However, in severe cases, with platelet counts of <20 000/µL, patients may experience bleeding complications such as intracranial and intestinal bleeding, which may lead to death [1]. ITP can be triggered by medication, infections, cancers, and autoimmune diseases [2,3].

Owing to the fact that ITP can be idiopathic and often presents with mild symptoms, it may not be diagnosed without extensive investigations [2]. Additionally, medications can cause thrombocytopenia, especially in older patients [3]. Therefore, it can be difficult to differentiate between medication-induced thrombocytopenia and ITP, particularly in older patients with multiple comorbidities receiving various drugs.

In our case, a rare presentation of ITP occurred following vaccination. There are various vaccines that have been associated with ITP, such as the pediatric measles and mumps vaccine [2,3]. However, influenza vaccine-related cases are rare, despite the common use of influenza vaccines worldwide. Here, we report a case of acute onset ITP following influenza vaccination. This is rare, and it is essential to differentiate ITP from thrombotic thrombocytopenia [2,3]. This case report provides a broader view of ITP and may facilitate the early detection of ITP following influenza vaccination.

## Case presentation

**Figure 1:**
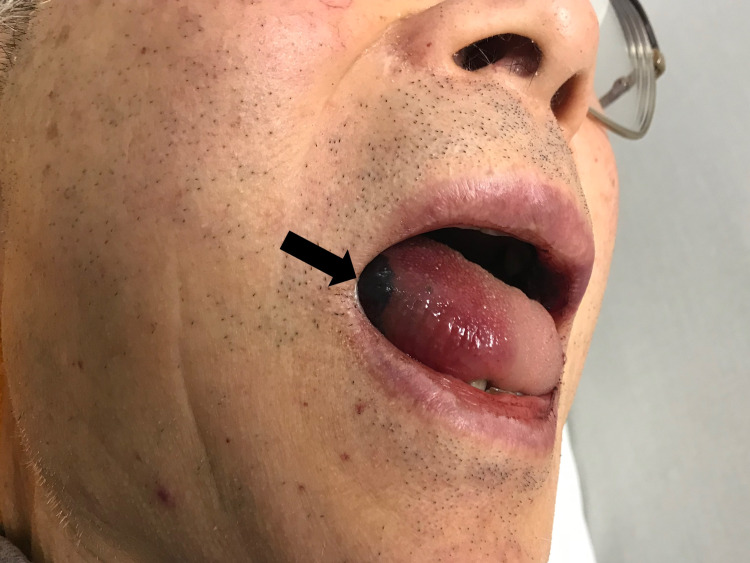
A bleeding blister noted on the left side of the tongue.

**Figure 2:**
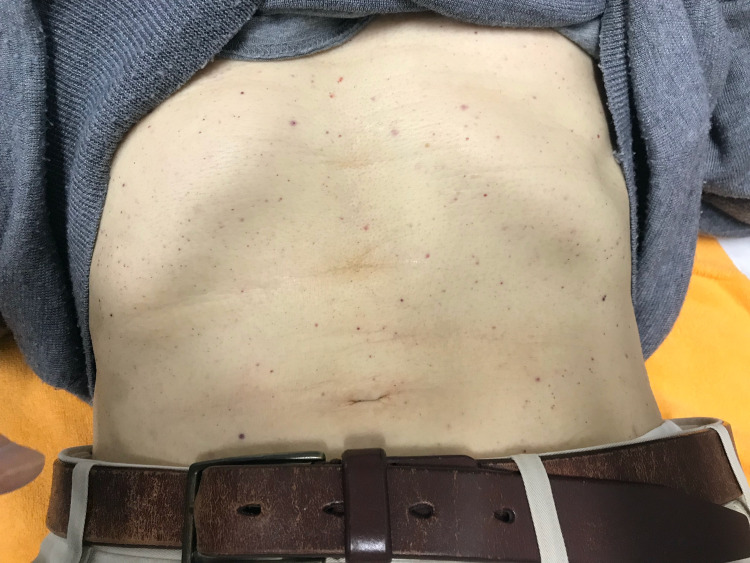
Multiple petechiae on the abdomen.

**Figure 3:**
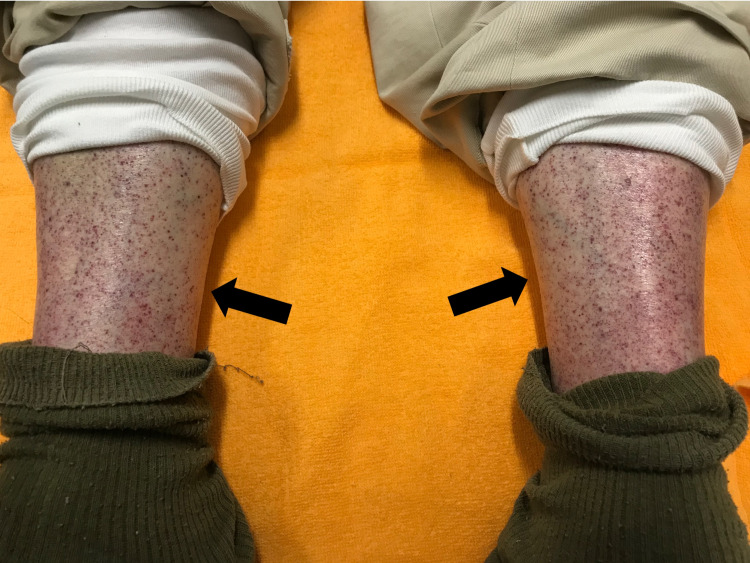
Multiple petechiae on the bilateral pretibial parts.

An 88-year-old man was admitted to our hospital with the chief complaint of bleeding from the mouth. Four days prior to admission, the patient had received an influenza vaccine injection. He did not experience any severe pain or anaphylactic symptoms. The patient had not experienced any infection or received any new medication in the last three months. His medical history included a brain stroke (15 years ago), hypertension, and dyslipidemia. He did not have any family history of ITP and other autoimmune diseases. The medications that he took included clopidogrel, valsartan, rosuvastatin, and esomeprazole. Physical examination revealed a bleeding blister on the left side of the tongue (Figure 1), and multiple petechiae on the surfaces of the abdomen (Figure 2) and anterior part of the lower legs (Figure 3).

No other abnormal findings were noted. Blood tests revealed the following: platelet count, 1000/µL; hemoglobin, 11.9 g/dL; lactate dehydrogenase, 228 IU/L; and glomerular filtration rate, 47.4 mL/min/1.73 m2. A month prior to presentation, a routine checkup in a primary care clinic had shown that the patient’s platelet count was 15.0×104/µL. Urine analysis revealed no proteinuria. The blood culture results were negative, and there were no schistocytes or fragmented red blood cells in the blood smears. A bone marrow biopsy revealed multiple megakaryocytes, without blastocytes or hemophagocytosis. Computed tomography from the head to the pelvis did not reveal any bleeding.

The patient was subsequently transferred to a tertiary hospital and diagnosed with influenza vaccine-induced severe ITP. He was treated with intravenous high-dose prednisolone and the symptoms disappeared with the platelet count recovering to >10.0×104/µL.

## Discussion

This case suggests that influenza vaccines can be associated with ITP as a rare complication. In vaccinated patients, vague symptoms such as general fatigue and itchiness may be an indicator to necessitate the examination of the skin and mucous membranes for signs of hemorrhage. 

Vaccines, including the influenza vaccine, can trigger ITP in rare cases. ITP is an autoimmune condition characterized by thrombocytopenia and is usually induced by viral infection [1]. Several large national studies from Italy and Israel have indicated that various vaccines, particularly the measles-mumps-rubella and diphtheria-tetanus-poliomyelitis vaccines, are associated with an increased risk of ITP [4,5]. Previous studies have shown that influenza vaccines can induce ITP [6]. However, although many case reports have been published on ITP triggered by vaccination in children, there are only a few reports of ITP related to influenza vaccination among adults [7,8]. ITP triggered by influenza vaccination should be considered In all age groups.

The possibility of ITP related to influenza vaccination should be considered in primary care settings. Influenza vaccines are the most commonly administered vaccines worldwide, with millions of people being vaccinated annually, especially in primary care settings [3]. There are a few reports of hematological side effects or thrombocytopenia resulting from this vaccine [7-9].

On the other hand, it is important to note that the risk of ITP related to the influenza vaccine is extremely low. A large study of vaccine-related adverse events in post-marketing surveillance from 1994 to 2004 in Japan reported only two cases of ITP among the recipients of 38 million doses of the influenza vaccine, along with three cases of acute disseminated encephalomyelitis and nine cases of Guillain-Barre syndrome [3,10]. However, despite the rarity of severe complications from influenza vaccination, family physicians should keep this possibility in mind during the vaccination season.

Based on the high annual prevalence of influenza vaccination and its proinflammatory effect [11], the median age of the patients in seven case reports describing influenza vaccine-induced ITP, including this case, was 65 years (interquartile range (IQR) 32-75), the median post-vaccine onset period was 14 days (IQR 7-15), and the median platelet count was 3000/µL (IQR 3000-10000). In addition, the symptoms suggestive of ITP may not be specific [2-4]. In this case, the initial symptoms were fatigue and itchiness. However, we were able to make a diagnosis based on the hemorrhagic findings. Previous studies have reported that influenza-induced ITP did not show any specific symptoms, except for petechiae and hemorrhage of the mucous membranes. However, severely low platelet counts (< 10000/µL) were present among some of the patients experiencing symptoms of itchiness and fatigue without hemorrhage [4,6]. In another report involving seven patients, in which all exhibited full recovery without sequelae, four were reported to have been treated with steroids and three with steroids and intravenous gamma globulin [6].

The ITP related to influenza vaccination may not cause mortality if adequate treatment is provided. Therefore older patients with the vaccination should be encouraged to observe their symptoms in order to not miss the symptoms of ITP. Older people should be informed regarding appropriate vaccinations of influenza and coronavirus disease 2019 (COVID-19) with adequate knowledge of the vaccination and its possible side effects. In the era of the COVID-19 pandemic, the importance of vaccination is to be emphasized [12-14]. Social media and various news resources can impinge older people towards being vaccinated, creating a vaccine phobia [12]. Social fear regarding COVID-19 can drive their vaccination especially in rural areas [15]. The improvement of their understanding of vaccines should not only improve the vaccination rates for COVID-19 but also for influenza. 

Furthermore, because of less movement of patients to other provinces, the comprehensiveness of medical care in rural areas is high [16]. This situation may need improvement of the primary care system regarding education and prevention of diseases among older patients. Rural older people tend to endure their symptoms because of the lack of help, causing the delay of diagnosis [17-19]. On the other hand, primary care is respected by them and used for various symptoms [19]. The role of primary care physicians can be vital for vaccination and follow-up of their symptoms. So, rural primary care physicians should drive present situations and educate older patients regarding the importance of vaccination and the correct timings to come and seek primary care, if necessary.

## Conclusions

Although vaccine-related ITP is rare, primary care physicians should perform a systematic physical examination to detect any hemorrhage in post-vaccination patients presenting with systemic symptoms to rule out ITP. In patients experiencing fatigue and itchiness following influenza vaccination, the diagnosis of ITP should be considered. ITP should also be considered in cases of suspected thrombocytopenia, and patients should be asked about their vaccination history.
